# Intergenerational associations between maternal health and offspring mental wellbeing: evidence from a nationally representative longitudinal study

**DOI:** 10.1007/s11136-026-04339-0

**Published:** 2026-07-23

**Authors:** Minnat Seema, Clifford Afoakwah, Joshua Byrnes

**Affiliations:** 1 Centre for Applied Health Economics (CAHE), Griffith University, Brisbane, QLD Australia; 2 Australian Centre for Health Services Innovation, School of Public Health and Social Work, Queensland University of Technology, Brisbane, Australia; 3 Jamieson Trauma Institute, Metro North Health, Brisbane, Australia

**Keywords:** Maternal mental health, Health-related quality of life, Intergenerational health, Longitudinal study, Public health, Social determinants of health

## Abstract

**Background:**

Maternal mental health has been linked to a range of offspring outcomes; however, population-based evidence examining intergenerational associations across multiple health-related quality of life (HRQoL) domains over the life course remains limited. This study examines associations between maternal mental health and offspring HRQoL using nationally representative longitudinal data from Australia.

**Methods:**

We used data from the Household, Income and Labour Dynamics in Australia (HILDA) Survey. The analytic sample comprised 8,737 offspring linked to 4,966 mothers. Three offspring’s HRQoL outcomes were examined: mental health and general health domain of the SF-36 instrument and the Kessler Psychological Distress Scale. Maternal mental health was measured using the SF-36 mental health domain. Associations were estimated using weighted linear regression models with standard errors clustered at the maternal level, adjusting for socioeconomic and demographic characteristics. Gender differences were examined using interaction terms.

**Results:**

Higher maternal mental health was significantly associated with better offspring mental health in fully adjusted models (β = 0.485 SD, *p* < 0.001). Higher maternal mental health was also associated with better offspring general health (β = 0.458 SD, *p* < 0.001). For psychological distress, higher maternal psychological distress was associated with higher offspring psychological distress (β = 0.419 SD, *p* < 0.001). Higher maternal mental health was also positively associated with offspring psychological distress (β = 0.216 SD, *p* < 0.001); however, this reflects differences in measurement constructs (higher distress scores indicate worse outcomes) and should be interpreted cautiously. Associations remained statistically significant after covariate adjustment and did not differ by offspring gender.

**Conclusions:**

Maternal mental health is associated with multiple dimensions of offspring HRQoL across the life course. These findings highlight maternal mental health as an important correlate of offspring health and underscore the relevance of family-centred, preventive strategies aimed at reducing intergenerational inequalities in mental health.

**Supplementary Information:**

The online version contains supplementary material available at https://doi.org/10.1007/s11136-026-04339-0.

## Background

Mental health problems account for a substantial and growing share of the global burden of disease and are a leading cause of disability across the life course [1]. Difficulties in mental health often emerge early in life and persist into adolescence and adulthood, shaping long-term trajectories in educational attainment, labour market participation, physical health, and overall health-related quality of life (HRQoL) [2, 3]. From a public health perspective, these patterns underscore the importance of identifying early-life determinants of mental health, and of implementing preventive strategies that reduce the risk of adverse trajectories before they become entrenched.

In this study, we examine the association between maternal mental health and the overall HRQoL of their offspring such as mental health, general health, and psychological distress. HRQoL refers to an individual’s perceived physical, mental, emotional and social wellbeing [4, 5]. Within this framework, mental health captures one’s perceived mental health status that can be measured using HRQoL instruments such as the 36-Item Short Form Survey (SF-36) and EuroQol-5 Dimension (EQ-5D), or disease-specific tools such as the Kessler Psychological Distress Scale that reflects symptoms of anxiety and depression. The SF-36 is one of the most widely used generic HRQoL instruments and measures eight domains of physical and mental health, providing a comprehensive assessment of overall health status. Although these constructs are related, they capture distinct but related dimensions of health and are therefore analysed separately in this study.

A substantial body of research demonstrates how maternal mental health is closely linked to children’s emotional, behavioural, and psychological development [6, 7]. Recent evidence also highlights the substantial burden of maternal mental health problems following disasters and the potential for long-term consequences for mothers and their children [8]. Offspring of mothers experiencing mental health difficulties face elevated risks of emotional and behavioural disorders, emotional dysregulation, and psychological distress across childhood and adolescence [9–11], outcomes that directly affect multiple domains of HRQoL. These associations are thought to reflect the combined influence of genetic vulnerability, prenatal stress biology, parenting practices, family environments, and shared socioeconomic conditions [12]. Understanding the strength and persistence of these intergenerational links is therefore central to population mental health and overall HRQoL research, as well as to the design of effective preventive interventions.

Despite extensive evidence documenting associations between maternal mental health and child outcomes, important gaps remain in population-representative longitudinal studies that explicitly frame these associations in terms of HRQoL. Much of the existing literature relies on cross-sectional data or short follow-up periods, limiting insight into how maternal health is associated with offspring mental health, general health, and psychological distress [13, 14]. Although recent longitudinal studies have demonstrated associations between persistent maternal mental health and offspring behavioural, academic, and educational outcomes [15], population-based evidence examining multiple dimensions of offspring HRQoL remains limited. In addition, many studies are unable to adequately account for unobserved family-level characteristics, such as stable psychosocial environments or long-standing socioeconomic disadvantage, that may confound observed associations between maternal and child health [16, 17]. These limitations restrict the capacity of public health systems to draw firm conclusions about the persistence and population-level significance of intergenerational inequalities in mental health and HRQoL.

Longitudinal, population-based cohort studies provide a valuable framework for addressing these challenges. Repeated observations within families allow for the examination of developmental timing, within-family patterns, and the consistency of intergenerational associations across multiple dimensions of HRQoL. Australia’s Household, Income and Labour Dynamics in Australia (HILDA) Survey offers a unique opportunity to advance this evidence base. HILDA is a nationally representative longitudinal panel study that collects repeated, validated measures of mental health, general health, and psychological distress, alongside detailed socioeconomic information. Its genealogical structure enables reliable linkage between children and their biological mothers over time, facilitating the construction of a large mother–child cohort.

The present study uses data from the HILDA Survey to examine intergenerational associations between maternal health and offspring mental health, while also assessing whether these associations extend to child general health and psychological distress, and whether patterns differ by child gender. Based on existing literature on intergenerational health transmission and maternal mental health, the study hypothesizes that better maternal mental health will be associated with better offspring mental health, better general health, and lower psychological distress in the long-term.

## Method

### Study design and reporting framework

This study employed an observational cohort design to examine intergenerational associations between maternal health and offspring HRQoL in a nationally representative Australian population. The analysis draws on longitudinal data from repeated survey waves but focuses on person-level health measures aggregated across available observations for each individual, capturing longer-run health profiles rather than within-wave dynamics. The study is reported in accordance with the Strengthening the Reporting of Observational Studies in Epidemiology (STROBE) guidelines for cohort studies [18], and a completed STROBE checklist is provided in the supplementary material.

## Data source

Data were obtained from the Household, Income and Labour Dynamics in Australia (HILDA) Survey, an ongoing nationally representative longitudinal household panel study that has followed Australian residents annually since 2001 [19]. HILDA collects detailed information on mental health, general health, psychological distress, socioeconomic circumstances, labour market participation, and family relationships using a combination of face-to-face interviews and self-completion questionnaires.

De-identified unit record data were accessed under licence via the Australian Data Archive. Although HILDA follows individuals over time, the present analysis utilises aggregated person-level measures derived from repeated observations across survey waves.

## Identification of mothers and offspring

Mother-offspring dyads were constructed using HILDA’s cross-wave genealogical identifiers, which link respondents to their biological parents across survey waves. A harmonised maternal identifier was created to ensure a stable and maternal identifier for each respondent over time. Offspring were identified using unique person-level panel identifiers.

To ensure a consistent intergenerational structure, each offspring was linked to a single maternal caregiver. For offspring linked to more than one maternal identifier across waves, the most frequently observed mother-offspring pairing was retained. This approach prioritised the most stable caregiving relationship observed across survey waves and minimised misclassification due to temporary household composition changes. Observations with missing maternal or offspring identifiers were excluded. This procedure yielded a unique maternal linkage for each offspring and allowed clustering at the maternal level to account for correlation between siblings. The HILDA genealogical identifiers primarily capture biological parent–child relationships; however, information distinguishing biological from non-biological maternal caregivers was not available in all cases. This is acknowledged as a limitation and may introduce some measurement uncertainty in intergenerational linkage.

## Participants and analytic sample

The analytic sample comprised mother-offspring dyads observed in at least one survey wave with valid health measures available for both the mother and the offspring. Health measures for each individual were averaged across all available survey waves to construct person-level indicators of longer-term HRQoL. Individuals were observed across multiple survey waves, reflecting the longitudinal nature of the HILDA Survey and enabling the construction of longer-run health measures.

After exclusions due to missing identifiers or health measures, 8,737 offspring linked to 4,966 unique mothers were eligible for analysis. Of these, 8,094 offspring were included in fully adjusted models with complete covariate information. The reduction in sample size in fully adjusted models reflects exclusion due to missing covariate data required for multivariable adjustment. Offspring were observed from childhood into adulthood, and the resulting sample reflects HRQol across the life course rather than specific developmental stages. Participant selection and exclusions are shown in Supplementary Figure S1.

## Outcome measures

Three proxies were used for offspring HRQoL outcomes: mental health, general health and psychological distress. Mental health and general health were measured respectively using the validated Mental Health Inventory-5 (MHI-5) and general health domains of the SF-36 instruments [20]. The SF-36 is a HRQoL instrument that consist of 36 questions and grouped into eight domains general health, mental health, physical function, bodily pain, role physical, vitality, social function, and role emotion. Because the instrument is generic rather than disease-specific, it enables comparisons across a range of diseases [21]. The SF-36 has demonstrated strong reliability and validity in Australian population studies using HILDA data, with reported Cronbach’s alpha values typically exceeding 0.80 [22, 23].

Mental health and general health scales range from 0 to 100, with higher scores indicating better health-related quality of life. To facilitate comparison across models and outcomes, these scores were standardised to z-scores. Psychological distress, on the other hand was measured using the Kessler Psychological Distress Scale [24], which has also demonstrated strong reliability and validity in Australian population studies [22, 23], and ranged from 10 to 50, with higher scores indicating greater psychological distress. All health measures were collected using self-completion questionnaires administered to individual respondents as part of the HILDA Survey. Offspring outcomes were self-reported rather than maternally reported, reducing the risk of same-informant bias. However, as with all self-reported measures, some degree of reporting bias cannot be ruled out. Also, all offspring health measures represent person-level averages across available survey waves.

### Exposure variable

The exposure variable in this study was maternal mental health measured using the MHI-5 from the same SF-36 instrument administered to adult respondents. Maternal mental health scores were averaged across available survey waves and standardised to z-scores. Parallel measures of maternal general health and psychological distress were examined in supplementary analyses to assess the robustness of associations across health domains.

## Covariates

Analyses adjusted for a comprehensive set of offspring and maternal characteristics selected a priori based on prior evidence linking sociodemographic characteristics, socioeconomic position, and health status to both parental health and offspring HRQoL (4, 5, 11). Offspring-level covariates included age, age squared, gender, educational attainment, employment status, disposable household income, and the presence of a long-term health condition. In addition, maternal socioeconomic position was included to account for shared family context and intergenerational transmission of advantage and disadvantage. These variables are commonly included in intergenerational and life-course studies using population-based cohort data to reduce confounding due to observed socioeconomic and demographic factors.

### Statistical analysis

Associations between maternal mental health score and offspring’s HRQoL were estimated using linear regression models. Health measures for mothers and offspring were averaged across all available survey waves to construct person-level indicators of longer-term mental health and general health. Aggregating health measures across waves allows us to capture typical or sustained health status, rather than short-term fluctuations. This approach is particularly appropriate for examining intergenerational associations, which are expected to reflect persistent family environments and exposures. However, this approach does not capture within-individual changes over time or developmental dynamics, which should be explored in future research. Accordingly, estimated associations reflect population-level relationships in long-term maternal and offspring health, rather than contemporaneous co-movement or dynamic causal effects.

For each outcome, a sequence of models was estimated to assess robustness to covariate adjustment and to examine potential gender differences. Initial models estimated unadjusted associations between maternal mental health score and offspring outcomes. Subsequent models adjusted for offspring-level and maternal socioeconomic and demographic characteristics. Additional models included an interaction term between maternal mental health score and offspring gender to assess whether associations differed between males and females. Equivalent model specifications were estimated for offspring general health and psychological distress outcomes.

To account for correlation between siblings, robust standard errors were clustered at the maternal level. All analyses incorporated survey weights to account for the complex sampling design of the HILDA Survey and to ensure population representativeness. Analyses were conducted using Stata version 15 (Stata Corp LLC, College Station, TX, USA), and statistical significance was assessed at the 5% level.

### Missing data

Missing values in maternal or offspring identifiers were addressed during data construction by excluding incomplete records prior to linkage. The reduction in sample size in fully adjusted models reflects missing data in covariates required for these specifications. Included and excluded participants were broadly similar across key observable characteristics, suggesting limited selection bias; however, the possibility of bias due to unobserved differences cannot be fully excluded. Models were estimated using all participants with available data for the variables included in each specification; sample sizes therefore vary across models and are reported in the Results tables. Participant selection and exclusions are shown in Supplementary Figure S1.

### Ethics

The HILDA Survey has received ongoing ethical approval from the Melbourne Institute Human Research Ethics Committee (initial approval granted in 2001 and renewed annually). The present study involved secondary analysis of fully de-identified unit record data and did not involve direct contact with participants.

In accordance with the Australian National Statement on Ethical Conduct in Human Research (2007, updated 2023), the Griffith University Human Research Ethics Committee determined that the project did not require separate ethical review or approval.

Access to HILDA data was granted to the authors under licence via the Australian Data Archive on 15 March 2024 (HILDA data access number 799488). All analyses were conducted in compliance with the HILDA data user agreement and relevant ethical standards.

## Results

### Sample characteristics

A total of 8,737 offspring linked to 4,966 unique mothers were eligible for analysis. The number of observations included in regression models varies across specifications due to missing outcome measures and covariate data.

The mean age of offspring was 28.4 years (SD = 11.1), spanning adolescence to adulthood. Approximately 49.7% were female, and 64.4% were employed, and 20.6% reported a long-term health condition. Mean levels of offspring and maternal health measure are presented in Table 1 with offspring having higher mental and general health scores than mothers. On average, each mother contributed 2.27 offspring (SD = 1.21) to the analytic sample. All descriptive statistics are weighted and account for clustering at the maternal level.

**Table 1:** Descriptive characteristics of the analytic sample

Variable	Mean (SD) or *n* (%)	*N*
Offspring mental health (MHI-5)	39.46 (26.88)	8,737
Maternal mental health (MHI-5)	33.12 (26.34)	8,737
Offspring general health (SF-36 GH)	49.00 (28.61)	8,737
Maternal general health (SF-36 GH)	30.11 (27.08)	8,737
Offspring psychological distress (Kessler)	7.99 (10.00)	7,882
Maternal psychological distress (Kessler)	5.61 (9.74)	7,412
Offspring age (years)	28.36 (11.10)	8,737
Maternal age (years)	29.7 (12.6)	4,966
Female	4,340 (49.7%)	8,737
Employed	5,626 (64.4%)	8,737
Years of education	6.22 (2.60)	8,124
Disposable household income	119,819 (112,883)	8,737
Long-term health condition	1,799 (20.6%)	8,737
Number of children per mother	2.27 (1.21)	8,737

Values are reported for the analytic mother–offspring sample. Continuous variables are presented as mean (standard deviation), and binary variables (female, employment, and long-term health condition) are presented as number (percentage)

Offspring and maternal mental health were measured using the SF-36 Mental Health Inventory (MHI-5), and general health using the SF-36 General Health (GH) scale. Higher MHI-5 and GH scores indicate better HRQoL

Psychological distress was measured using the Kessler Psychological Distress Scale. Reported values reflect aggregated person-level averages across available survey waves and therefore do not correspond directly to the original 10–50 scale range. Higher values indicate greater psychological distress

Sample sizes vary across variables due to missing data. Maternal age represents the average age across observed survey waves, rather than age at childbirth

All statistics are weighted using person-level population weights from the HILDA Survey, and standard errors are clustered at the maternal level to account for correlation between siblings

### Association between maternal mental health and offspring mental health

Table 2 reports associations between maternal mental health and offspring mental health across a sequence of weighted regression models. In unadjusted models, higher maternal mental health score was strongly and positively associated with better offspring mental health (β = 0.583 SD, SE = 0.004). After adjustment for offspring age, age squared, gender, education, employment status, disposable household income, long-term health conditions, and maternal socioeconomic characteristics, the magnitude of the association was attenuated but remained statistically significant (β = 0.485 SD, SE = 0.007), indicating that the association persists beyond observed socioeconomic and demographic factors. Several covariates were also significantly associated with offspring mental health. Lower educational attainment, lower household income, and the presence of a long-term health condition were consistently associated with poorer offspring mental health. Offspring age exhibited a non-linear association with mental health (Table 2).

**Table 2:** Association between maternal mental health and offspring mental HRQoL

	Model 1	Model 2	Model 3	Model 4
Maternal mental health (standardised)	0.583*** (0.004)	0.485*** (0.007)	0.578*** (0.006)	0.480*** (0.009)
Female offspring (ref: male)			0.025* (0.011)	0.008 (0.011)
Maternal mental health × Female offspring			0.011 (0.009)	0.010 (0.009)
Offspring socioeconomic factors	No	Yes	No	Yes
Maternal socioeconomic factors	No	Yes	No	Yes
Age and age squared	No	Yes	No	Yes
Observations (N)	8,737	8,094	8,737	8,094
Number of mothers	4,966	4,746	4,966	4,746

The outcome is offspring mental health, measured using the SF-36 Mental Health Inventory (MHI-5) and standardised to z-scores. Maternal mental health is also standardised to z-scores. Regression coefficients represent standard deviation (SD) changes in offspring mental health associated with a one SD increase in maternal mental health

Model 1 estimates unadjusted associations. Model 2 adjusts for offspring and maternal socioeconomic and demographic characteristics, including education, employment status, disposable household income, long-term health conditions, age (and age squared). Model 3 extends Model 1 by including an interaction term between maternal mental health and offspring gender. Model 4 extends Model 2 by including the interaction term

All models are estimated using person-level population weights, with robust standard errors clustered at the maternal level. Male offspring is the reference category

*p < 0.05; **p < 0.01; ***p < 0.001

### Gender differences in intergenerational associations

Models including interaction terms between maternal mental health and offspring gender are also presented in Table 2. Female offspring exhibited slightly higher mental health than males in unadjusted models (β = 0.025 SD, SE = 0.011), although this difference was not statistically significant after covariate adjustment (β = 0.008 SD, SE = 0.011).

The interaction between maternal mental health and offspring gender was not statistically significant in either unadjusted (β = 0.011 SD, SE = 0.009) or adjusted models (β = 0.010 SD, SE = 0.009), indicating no evidence that the association between maternal and offspring mental health differs by gender.

### Maternal mental health and child general health and psychological distress

Table 3 extends the analysis to additional offspring health outcomes. In fully adjusted models, higher maternal general health score was associated with better offspring general health (β = 0.488 SD, SE = 0.008). Higher maternal mental health score was also associated with better offspring general health (β = 0.458 SD, SE = 0.009).

**Table 3:** Associations between maternal health–related quality of life and offspring HRQoL outcomes

	Offspring general health	Offspring psychological distress
Maternal general health (standardised)	0.488*** (0.008)	–
Maternal mental health (standardised)	0.458*** (0.009)	0.216*** (0.013)
Maternal psychological distress (standardised)	–	0.419*** (0.012)
Female offspring (ref: male)	0.019 (0.012)	0.125*** (0.014)
Maternal mental health × Female offspring	–	− 0.016 (0.016)
Offspring socioeconomic factors	Yes	Yes
Maternal socioeconomic factors	Yes	Yes
Offspring age and age squared	Yes	Yes
Observations (N)	6,087	5,085
Number of mothers	3,797	3,060

Outcomes are standardised (z-scores). Offspring general health is measured using the SF-36 General Health (GH) scale, where higher scores indicate better health, while psychological distress is measured using the Kessler Psychological Distress Scale, where higher scores indicate greater distress. Maternal health variables are standardised and correspond to the same or alternative maternal measures as specified

Regression coefficients represent standard deviation (SD) changes in offspring outcomes associated with a one SD increase in maternal health. All models adjust for offspring and maternal socioeconomic and demographic characteristics, including education, employment status, disposable household income, long-term health conditions, and offspring age (and age squared). Models are estimated using person-level population weights, with robust standard errors clustered at the maternal level. Male offspring is the reference category

Interaction between maternal mental health and offspring gender was additionally estimated for psychological distress

*p < 0.05; **p < 0.01; ***p < 0.001

For psychological distress, higher maternal psychological distress was associated with higher offspring psychological distress (β = 0.419 SD, SE = 0.012). Higher maternal mental health score was also positively associated with offspring psychological distress (β = 0.216 SD, SE = 0.013). This pattern likely reflects differences in measurement constructs and standardisation across health domains rather than a direct protective relationship and should therefore be interpreted with caution.

Female offspring reported higher levels of psychological distress than males (β = 0.125 SD, SE = 0.014), while no statistically significant gender differences were observed for general health (β = 0.019 SD, SE = 0.012). Consistent with findings for mental health, lower socioeconomic position and the presence of long-term health conditions were associated with poorer offspring health outcomes across models. An interaction model (Table 3) showed no significant gender difference in the association between maternal mental health and offspring psychological distress (β = -0.016, SE = 0.016, *p* = 0.321).

### Summary of findings

Across multiple outcome measures, maternal health, particularly maternal mental health, was consistently associated with offspring mental health, general health, and psychological distress. These associations remained robust after adjustment for a wide range of socioeconomic, demographic, and health-related factors and were observed across multiple health domains. Overall, the findings highlight maternal mental and general health as key correlates of offspring HRQoL outcomes across the life course at the population level.

## Discussion

### Principal findings

Using nationally representative longitudinal data from Australia, this study provides consistent evidence of statistically significant intergenerational associations between maternal mental and offspring HRQoL across multiple health domains. Lower maternal mental health was associated with lower offspring mental health, poorer general health, and higher levels of psychological distress. These associations persisted after adjustment for a wide range of socioeconomic, demographic, and health-related factors, indicating that maternal mental and general health remains associated with offspring HRQoL across adolescence and early adulthood.

The consistency of associations across alternative outcome measures, including mental health, general health, and psychological distress (Kessler), suggests that maternal mental health is linked not only to offspring mental health but also to broader dimensions of the offsprings’ overall HRQoL. This pattern underscores the interconnected nature of mental and general health within family contexts and highlights maternal wellbeing as a relevant correlate of offspring HRQoL beyond narrowly defined psychiatric outcomes.

### Comparison with existing literature

These findings align with and extend a substantial body of literature documenting associations between parental mental health and offspring psychological outcomes ([22]; [6, 7, 10]).

Previous studies, often based on cross-sectional data or short follow-up periods, have reported links between maternal psychological distress and offspring internalising and externalising problems. By leveraging the longitudinal structure of the HILDA Survey and repeated observations of linked mother–offspring dyads, this study strengthens existing evidence by demonstrating that these associations persist when maternal and offspring mental health, general health, and psychological distress are measured as longer-term averages derived from repeated observations and across several validated health measures. This study contributes to the literature in three ways. First, the use of multiple validated HRQoL outcome measures reduces the likelihood that findings are driven by scale-specific reporting behaviour. Second, adjustment for a comprehensive set of socioeconomic and health-related covariates allows for a more nuanced assessment of the extent to which associations reflect shared family circumstances. Third, the absence of statistically significant gender differences suggests that intergenerational associations in HRQoL operate similarly for male and female offspring, reinforcing the broad relevance of maternal mental health for offspring HRQoL across genders. This finding is consistent with some studies reporting limited or no gender differences in intergenerational mental health associations, although other research has identified stronger associations among female offspring in certain contexts (7). These mixed findings highlight the importance of population-based longitudinal analyses in clarifying the consistency of intergenerational patterns across genders.

### Interpretation and potential mechanisms

The observed intergenerational associations likely reflect a combination of psychosocial, environmental, and biological pathways. Maternal mental health may influence offspring overall HRQoL through stress-related processes, emotional availability, parenting practices, and the overall psychosocial climate of the household [22]. Shared socioeconomic conditions and health environments may also contribute to correlated health trajectories within families.

Although the use of longitudinal data strengthens population-level inference, the findings should be interpreted as associations rather than causal effects. Time-varying unobserved factors, such as acute family stressors or changes in household circumstances, may influence both maternal and offspring health within the same period. Nevertheless, the consistency of associations across outcomes and model specifications suggests that maternal mental health represents a salient and policy-relevant marker of risk for poorer offspring HRQoL at the population level. These pathways are consistent with prior research highlighting the role of family environment, parenting practices, and shared socioeconomic conditions in shaping intergenerational mental health outcomes [6, 7].

#### Public health and service delivery implications

From a public health perspective, these findings highlight maternal mental health as an important component of family-centred prevention strategies. Because maternal mental health is associated with multiple dimensions of offspring HRQoL, interventions that support maternal mental health may yield benefits that extend beyond individual outcomes and influence broader population health trajectories.

The results suggest that policies and services focused solely on short-term symptom management may have limited intergenerational impact unless they contribute to sustained improvements in maternal mental health. Public health strategies that emphasise early identification, continuity of care, and integrated family-based support may therefore play a critical role in mitigating the intergenerational persistence of poorer mental health and related health outcomes.

These findings are particularly relevant for community-based and primary care services operating at the interface of adult and youth mental health.

### Policy implications

At a systems level, the findings underscore the potential population-level gains associated with sustained investment in maternal mental health. Given the associations observed across mental health, general health, and psychological distress, policies aimed at improving maternal mental health may contribute to broader improvements in offspring HRQoL and reductions in long-term mental health inequalities.

These results support approaches that embed maternal mental health within life-course and family-oriented public health frameworks. Addressing maternal mental health as part of preventive policy initiatives may therefore yield benefits that extend across generations.

#### Strengths and limitations

This study has several strengths, including the use of a large, nationally representative longitudinal dataset, validated HRQoL and related health measures, and detailed socioeconomic information. The linkage of offspring to biological mothers and the aggregation of repeated observations enhance the robustness of the analysis and allow for assessment of intergenerational associations across multiple health domains.

However, some limitations should also be acknowledged. Health measures were based on self-reported survey instruments and may be subject to reporting bias. Although multiple outcomes were examined, measurement error cannot be fully excluded. The observational design limits causal inference, and time-varying unobserved factors may influence both maternal and offspring health outcomes. While excluded participants were broadly similar to those included in terms of age, income, and socioeconomic position, males were slightly over-represented among excluded participants. Some analyses relied on complete-case data due to missing covariate information. In addition, the data do not consistently distinguish between biological and non-biological maternal caregivers, which may introduce heterogeneity in intergenerational associations. Finally, although the HILDA Survey is broadly representative of the Australian household population, findings may not generalise to institutionalised populations or families experiencing severe disadvantage who are under-represented in longitudinal surveys.

## Conclusions

This study provides longitudinal, population-level evidence of intergenerational associations between maternal mental health and offspring HRQoL across multiple domains. The findings highlight maternal mental health as a key correlate of offspring HRQoL and underscore the importance of family-centred, preventive approaches within public health systems. By demonstrating that maternal mental health is linked to both offspring mental health and broader health outcomes, this study supports integrated strategies aimed at improving HRQoL and related health outcomes across generations.

## Supplementary Information

Below is the link to the electronic supplementary material.

Supplementary Material 1

Supplementary Material 2

## Data Availability

The data that support the findings of this study are available from the Australian Data Archive; however, restrictions apply to their availability, as the data are provided under licence. Researchers may apply for access to the HILDA Survey data through the Australian Data Archive, subject to approval by the data custodians.
