# Supplementary material for: Intergenerational associations between maternal health and offspring mental wellbeing: evidence from a nationally representative longitudinal study
Source: Qual Life Res. 2026 Jul 23;35(9):232. doi: 10.1007/s11136-026-04339-0 (PMC13395870; doi:10.1007/s11136-026-04339-0)
Supplement: Supplementary file 1 — Supplementary Material 1 [file 11136_2026_4339_MOESM1_ESM.docx]

**Supplementary file #2**

STROBE Statement—Checklist of items that should be included in reports of ***cohort studies***

**Title: Intergenerational associations between maternal health and child mental wellbeing: evidence from a nationally representative longitudinal study**

|  | Item No | Recommendation |
| --- | --- | --- |
| **Title and abstract** | 1 | (*a*) The title and abstract explicitly describe the study as a national longitudinal cohort study using the HILDA Survey. |
|  |  | (*b*) The abstract provides a balanced summary of the data source (HILDA), study population (linked mother–child cohort), exposures and outcomes, statistical methods (weighted longitudinal regression with clustered standard errors), and the direction and magnitude of key findings. |
| Introduction | | |
| Background/rationale | 2 | The introduction describes the scientific background linking maternal health to child mental wellbeing, highlights its relevance to population mental health, quality of life, and preventive public health policy, and identifies gaps in existing evidence related to longitudinal timing and unobserved family heterogeneity. |
| Objectives | 3 | The study objectives are clearly stated: to examine intergenerational associations between maternal health and child mental wellbeing using longitudinal data, to assess robustness across multiple mental health outcomes, and to explore potential gender-based differences. |
| Methods | | |
| Study design | 4 | This study is an observational longitudinal cohort analysis using secondary data from the Household, Income and Labour Dynamics in Australia (HILDA) Survey. |
| Setting | 5 | The study setting is Australia, using nationally representative household survey data collected annually between 2001 and 2022 with repeated follow-up of participants across waves. |
| Participants | 6 | (*a*) Eligible participants included children and young people with valid mental health measures who could be linked to their biological mothers using the HILDA genealogical file and who had valid survey weights. Participants contributed repeated observations across survey waves. |
|  |  | (*b*) Not applicable; this study did not use matching. |
| Variables | 7 | Maternal health measures were the primary exposures. Child mental wellbeing outcomes included validated measures of mental health and psychological distress. Covariates included child and maternal socioeconomic status, education, employment, income, long-term illness, age, and gender. Child gender was examined as a potential effect modifier. |
| Data sources/ measurement | 8* | All variables were derived from validated HILDA Survey instruments, including SF-36 mental health items and the Kessler psychological distress scale. Mother–child linkages were established using HILDA genealogical identifiers, with consistent measurement methods across survey waves*.* |
| Bias | 9 | Potential sources of bias were addressed through the use of validated instruments, survey weights to account for non-response, exclusion of invalid response categories, multivariable adjustment for confounding, and clustering of standard errors at the maternal level, while acknowledging the possibility of residual confounding. |
| Study size | 10 | The study size was determined by the number of linked mother–child observations meeting inclusion criteria after data cleaning and availability of valid outcome, exposure, and covariate information. |
| Quantitative variables | 11 | Continuous exposure and outcome variables were standardised (z-scores) to facilitate comparability across measures. Age was modelled flexibly using linear and quadratic terms. |
| Statistical methods | 12 | (*a*) Associations were estimated using weighted linear regression models with robust standard errors clustered at the maternal level to account for repeated observations and within-family correlation. |
|  |  | (*b*) Observations with invalid or non-response codes were excluded. Analyses were conducted using complete cases for each model, and the resulting sample sizes are reported accordingly. |
|  |  | (*c*) Observations with invalid or non-response codes were excluded. Analyses were conducted using complete cases for each model, and sample sizes are reported accordingly. |
|  |  | (*d*) The HILDA Survey is an unbalanced longitudinal panel in which participants may enter and exit across waves. This was accounted for through the use of survey weights and appropriate longitudinal estimation; no explicit attrition modelling was undertaken.” |
|  |  | (*e*) Sensitivity analyses included alternative mental health outcome measures and model specifications with and without covariate adjustment. |
| Results | | |
| Participants | 13* | (a) The number of observations at each stage of data cleaning and analysis is reported, including the final analytic sample for each outcome |
|  |  | (b) Reasons for exclusion included missing linkage, invalid survey responses, and missing outcome or exposure data. |
|  |  | (c) A flow diagram was not included due to the use of secondary longitudinal survey data with repeated observations across waves. |
| Descriptive data | 14* | (a) Descriptive characteristics of children and mothers, including health measures and socioeconomic variables, are presented. |
|  |  | (b) The number of observations available for each variable is reported in descriptive tables and model sample sizes. |
|  |  | (c) Participants contributed repeated observations across multiple survey waves, reflecting longitudinal follow-up. |
| Outcome data | 15* | Summary statistics for child mental wellbeing, general health, and psychological distress outcomes are reported using descriptive tables and model-specific sample sizes. |
| Main results | 16 | (*a*) Unadjusted and adjusted regression estimates are presented with 95% confidence intervals, with confounders specified. |
|  |  | (*b*) Not applicable; continuous variables were analysed without categorisation. |
|  |  | (*c*) Not applicable; outcomes were analysed on continuous mental health scales. |
| Other analyses | 17 | Additional analyses examined gender interactions and robustness across alternative outcome measures. |
| Discussion | | |
| Key results | 18 | Key findings are summarised in relation to the study objectives. |
| Limitations | 19 | Study limitations, including residual confounding, self-reported measures, and panel attrition, are discussed. |
| Interpretation | 20 | Results are interpreted cautiously in light of the study design, existing literature, and implications for population mental health, quality of life, and preventive public health strategies |
| Generalisability | 21 | Findings are generalisable to the Australian household population, with limitations for non-household and institutionalised populations. |
| Other information | | |
| Funding | 22 | This study did not receive any specific external funding. |

*Give information separately for exposed and unexposed groups.

**Note:** An Explanation and Elaboration article discusses each checklist item and gives methodological background and published examples of transparent reporting. The STROBE checklist is best used in conjunction with this article (freely available on the Web sites of PLoS Medicine at http://www.plosmedicine.org/, Annals of Internal Medicine at http://www.annals.org/, and Epidemiology at http://www.epidem.com/). Information on the STROBE Initiative is available at http://www.strobe-statement.org.
