# Supplementary material for: Intergenerational associations between maternal health and offspring mental wellbeing: evidence from a nationally representative longitudinal study
Source: Qual Life Res. 2026 Jul 23;35(9):232. doi: 10.1007/s11136-026-04339-0 (PMC13395870; doi:10.1007/s11136-026-04339-0)
Supplement: Supplementary file 2 — Supplementary Material 2 [file 11136_2026_4339_MOESM2_ESM.docx]

**Supplementary File #1**

**Supplementary Material**

**Supplementary Figure S1. Flow diagram of participant selection**
The figure illustrates the construction of the analytic sample from the Household, Income and Labour Dynamics in Australia (HILDA) Survey. Offspring were linked to their biological mothers using cross-wave genealogical identifiers. The analytic sample includes offspring with valid maternal linkage and available health measures. Exclusions reflect missing covariate information required for fully adjusted models. Final sample sizes correspond to those reported in the main analyses.


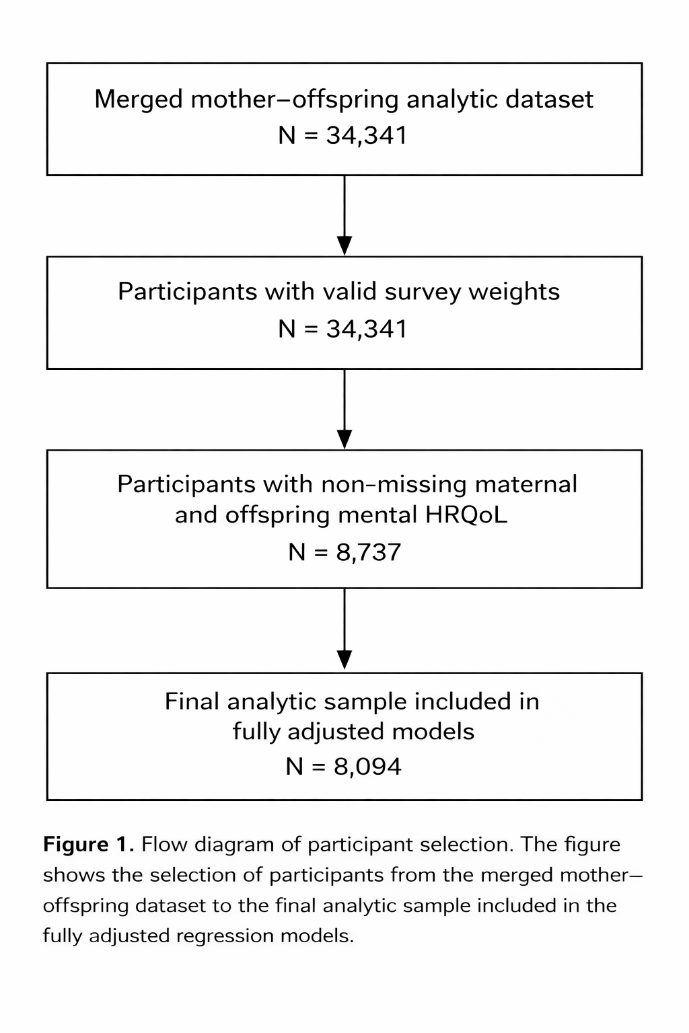


**Supplementary Figure S1. Flow diagram of participant selection.**
